# Single-Cell Transcriptomics Analysis of Human Small Antral Follicles

**DOI:** 10.3390/ijms222111955

**Published:** 2021-11-04

**Authors:** Xueying Fan, Ioannis Moustakas, Monika Bialecka, Julieta S. del Valle, Arend W. Overeem, Leoni A. Louwe, Gonneke S. K. Pilgram, Lucette A. J. van der Westerlaken, Hailiang Mei, Susana M. Chuva de Sousa Lopes

**Affiliations:** 1 Department of Anatomy and Embryology, Leiden University Medical Center, 2333 ZC Leiden, The Netherlands; x.fan@lumc.nl (X.F.); I.Moustakas@lumc.nl (I.M.); m.bialecka-dejong@uu.nl (M.B.); J.S.del_Valle@lumc.nl (J.S.d.V.); A.W.Overeem@lumc.nl (A.W.O.); 2 Sequencing Analysis Support Core, Department of Biomedical Data Sciences, Leiden University Medical Center, 2333 ZC Leiden, The Netherlands; H.Mei@lumc.nl; 3 Department of Gynaecology, Leiden University Medical Center, 2333 ZA Leiden, The Netherlands; L.A.Louwe@lumc.nl (L.A.L.); G.S.K.Pilgram@lumc.nl (G.S.K.P.); L.A.J.van_der_Westerlaken@lumc.nl (L.A.J.v.d.W.); 4 Department for Reproductive Medicine, Ghent University Hospital, 9000 Ghent, Belgium

**Keywords:** human adult ovary, oocyte, granulosa cell, antral follicle, single-cell transcriptomics, signaling pathways

## Abstract

Human ovarian folliculogenesis is a highly regulated and complex process. Characterization of follicular cell signatures during this dynamic process is important to understand follicle fate (to grow, become dominant, or undergo atresia). The transcriptional signature of human oocytes and granulosa cells (GCs) in early-growing and ovulatory follicles have been previously described; however, that of oocytes with surrounding GCs in small antral follicles have not been studied yet. Here, we have generated a unique dataset of single-cell transcriptomics (SmartSeq2) consisting of the oocyte with surrounding GCs from several individual (non-dominant) small antral follicles isolated from adult human ovaries. We have identified two main types of (healthy) follicles, with a distinct oocyte and GC signature. Using the CellphoneDB algorithm, we then investigated the bi-directional ligand–receptor interactions regarding the transforming growth factor-β (TGFβ)/bone morphogenetic protein (BMP), wingless-type (MMTV)-integration site (WNT), NOTCH, and receptor tyrosine kinases (RTK) signaling pathways between oocyte and GCs within each antral follicle type. Our work not only revealed the diversity of small antral follicles, but also contributes to fill the gap in mapping the molecular landscape of human folliculogenesis and oogenesis.

## 1. Introduction

Human ovarian folliculogenesis represents one of the most complex biological processes within the body and protocols to recapitulate folliculogenesis in vitro in clinical settings are highly desirable for fertility preservation purposes [1,2]. Its complexity is reflected in the balance between growth and remodeling of the ovarian follicles, but also on the high level of synchronization in the different follicular cell types within one follicle with the common goal of producing a competent mature oocyte [3].

In the human ovary, the generation of primordial ovarian follicles, consisting of an oocyte arrested at the diplotene stage (dictyate) of meiotic prophase I surrounded by a single layer of flat (or squamous) granulosa cells (GCs) occurs before birth [4,5]. Subsequently, a pool of primordial follicles will become active, the layer of GCs surrounding the oocyte will change morphology from flat to cuboidal and the oocyte will start increasing in size. The GCs may start to proliferate, forming several layers around the oocyte, forming secondary follicles (<0.15 mm) and pre-antral follicles (0.15–0.2 mm). The GCs will allow accumulation of the follicular fluid to a central growing cavity, known as antrum, giving rise to growing antral follicles [6]. Antral follicles between 2–5 mm can be detected in the ovary throughout the menstrual cycle, but in mono-ovulatory species, including humans, typically only one follicle becomes the dominant, reaching about 20 mm in diameter by the time of ovulation [7]. The other antral follicles will undergo atresia during this competitive selection [8]. Atretic follicles are often observed in a cohort of antral follicles, recognized by morphological changes, which include GC detachment from the basement membrane, macrophage infiltration, death of GCs, and luteinization of theca cells [9,10]. To date, factors that determine follicle fate (dominance or atresia) are still not well understood [7,11].

Two main subtypes of GCs are present in antral follicles: the cumulus GC (surround and provide support to the oocyte) and the mural GCs (line and provide mechanical support to the follicular wall and have a steroidogenic function) [9,12]. Although studies on non-human species report that oocyte-secreted factors play a role inducing differentiation to cumulus GCs [13,14], human cumulus GCs have mostly been studied at the ovulatory stage as tool to predict oocyte competence [15] and less is known about oocyte-cumulus GCs crosstalk in the context of antral follicle growth.

Based on the maturation state of the nucleus, oocytes can be classified into germinal vesicle (GV), metaphase I (MI), and metaphase II (MII). It is the peak of luteinizing hormone (LH), produced by the pituitary, that allows the GV oocyte (arrested in dictyate) to undergo GV breakdown, during which the nuclear envelope disintegrates. The oocyte then resumes meiosis, proceeds through the MI stage, extrudes the first polar body (PB) and arrests in the MII stage until fertilization takes place [16]. Studies on human GV oocytes from early/small antral follicles revealed heterogeneous cellular features regarding chromatin configuration, transcriptional activity, and mitochondria rearrangements [17]. However, whether this heterogeneity reflects different molecular signatures remains unclear.

Recently, we and others have used single-cell transcriptomics to reveal the molecular landscape of human ovarian follicular cells from different growing stages [9,18,19,20]. However, the analysis of oocyte and surrounding GCs from individual antral follicles has not been performed. Due to the multistep nature of folliculogenesis, it is important to investigate the relationship between oocyte and GCs within the same (growing or degenerating) follicle. Here, we have isolated single cells from several antral follicles and provide a transcriptional analysis of the oocyte and associated GCs. This work aimed to characterize the transcriptional signatures of different individual antral follicles (including the oocyte and associated GCs) and offers insights in the regulation of antral follicle growth in humans.

## 2. Results

### 2.1. Single-Cell RNA Sequencing of Human Oocytes and Follicular Somatic Cells

**Figure 1:**
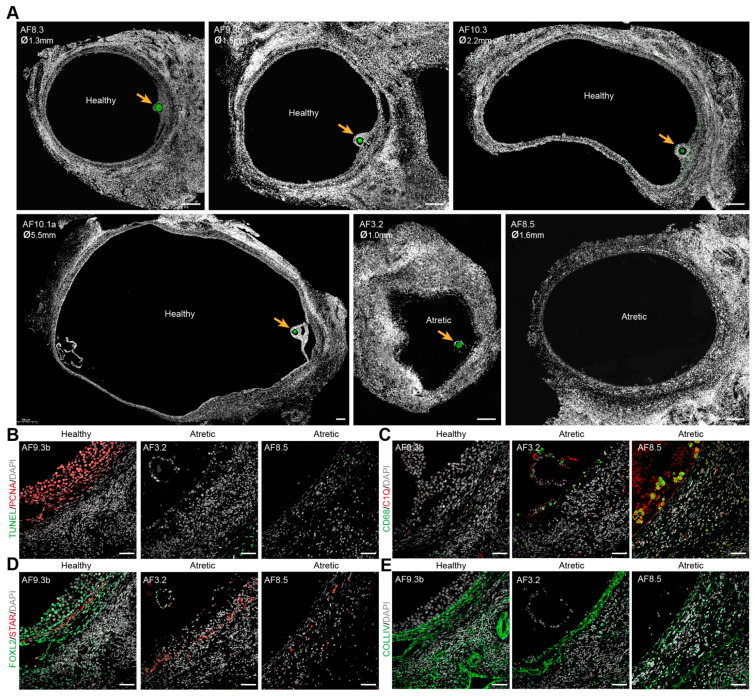
Morphology of healthy and atretic small antral follicles in adult ovaries. (**A**) Immunofluorescence for DDX4 (green) in healthy and atretic small antral follicles (AF). Orange arrows indicate DDX4+ oocytes. ø, follicle diameter in millimeters (mm); (**B**) Immunofluorescence for PCNA and TUNEL staining showing cell proliferation (PCNA+) and cell apoptosis (TUNEL+) in healthy and atretic follicles; (**C**) Immunofluorescence for CD68 and C1Q showing localization of macrophages (CD68+C1Q+) in healthy and atretic follicles; (**D**) Immunofluorescence for FOXL2 and STAR showing (FOXL2+) GCs, (FOXL2+) non-steroidogenic theca cells (TC) and (STAR+) steroidogenic TC in healthy and atretic follicles; (**E**) Immunofluorescence for COLIV marking the basement membrane. Scale bars in (**A**) are 200 μm and in (**B**–**E**) are 50 μm.

**Figure 2:**
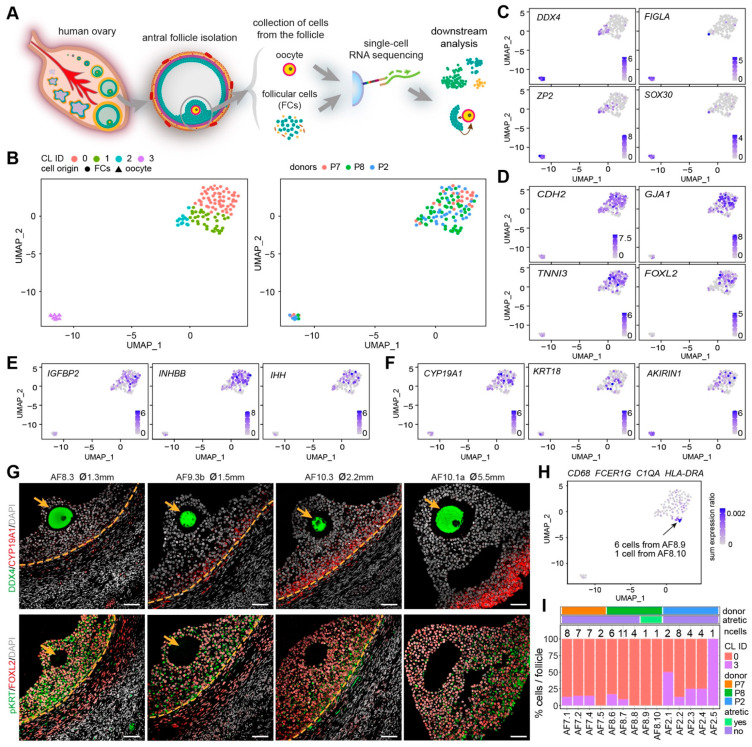
Identification of oocytes and granulosa cells from small human antral follicles. (**A**) Schematic illustration of the study design; (**B**) Uniform manifold approximation and projection (UMAP) plots showing 4 clusters of cells (left panel), colored by donors (right panel); (**C**) Expression of oocyte markers on the UMAP plot; (**D**) Expression of general granulosa cell (GCs) markers on the UMAP plot; (**E**) Expression of cumulus GC markers on the UMAP plot; (**F**) Expression of mural GC markers on the UMAP plot; (**G**) Immunofluorescence for DDX4 and CYP19A1 (top panels); and pan-cytokeratin (pKRT) and FOXL2 (bottom panels) on small human antral follicles showing the follicle area close to the oocyte. Orange arrows indicate the oocyte. Orange dashed lines indicate the follicular basement membrane. ø, follicle diameter. Scale bars are 50 μm; (**H**) Combined expression of selected immune markers on the UMAP plot; (**I**) Percentage of oocytes (CL3) and GCs (CL0) per small antral follicle.

We have collected the ovarian inner cortex/medulla tissue from female donors (*n* = 6) undergoing fertility preservation (Supplementary Figure S1A) and isolated small antral follicles (healthy or atretic) (Figure 1 and Supplementary Figure S1B). For single-cell sequencing, from each isolated follicle, the cumulus part containing GCs and oocyte were trimmed off and single cells were collected (Figure 2A). A total of 141 cells from 14 individual antral follicles (5 from donor P2; 4 from donor P7; 5 from donor P8) were sent for single-cell RNA sequencing (SmartSeq2). After quality control, 113 cells, including 10 oocytes and 103 follicular somatic cells, were retained for downstream analysis and used for cell clustering based on a Seurat-based workflow [21]. After correction for batch effect, four main clusters (CL) were identified by uniform manifold approximation and projection (UMAP) analysis, and each contained cells from different donors (Figure 2B) and follicles (Supplementary Figure S2A). Differentially expressed genes were calculated for each cluster (Supplementary Table S1). The collected oocytes clustered in CL3 and expressed oocyte markers, such as *DDX4*, *FIGLA*, *ZP2,* and *SOX30* (Figure 2C). According to known markers for the main types of human ovarian somatic cells [9,19,20], cells in CL0 were GCs, showing high expression of GC specific markers, such as *CDH2*, *GJA1*, *TNNI3,* and *FOXL2* (Figure 2D); and ovarian stromal (and theca) cells recognized by expression of *DCN*, *APOE*, *COL1A1* and *COL3A1* were present in CL1 and CL2 (Supplementary Figure S2B). Cells in CL2 showed expression of endothelial marker genes (*VWF* and *CD34*) and smooth muscle cell markers (*TAGLN* and *RGS5*), indicating that cells in CL2 may include endothelial and perivascular cells (Supplementary Figure S2C).

Surprisingly, the GCs in our dataset (CL0) showed no separation between cumulus GCs (*IGFBP2*, *INHBB*, *IHH*) and mural GCs (*CYP19A1*, *KRT18*, *AKIRIN1*) [9,22,23] (Figure 2E,F), suggesting that the mural GCs near the cumulus area may still share a molecular signature with cumulus GCs in early antral follicles. To further understand how cumulus and mural GC distributed near the oocyte, we performed immunostaining for mural GC markers, CYP19A1 and pan-cytokeratin (pKRT) (Figure 2G and Supplementary Figure S2D). We observed that CYP19A1 was expressed in mural GCs, but gradually upregulated in mural GC in the cumulus area (Figure 2G and Supplementary Figure S2D). By contrast, pKRT was initially uniformly expressed in all GCs (mural and cumulus); however, it became restricted to mural GCs and also those present near the cumulus (Figure 2G and Supplementary Figure S2D). We conclude that the GCs, both cumulus and mural, located near the cumulus in small antral follicles are still undergoing differentiation and that further analysis of the GCs in the cumulus area is needed to fully characterize different GC populations. 

We detected a group of cells in CL1 that showed high expression of *FCER1G* and *CD68* (Supplementary Figure S2E), characteristic of immune cells. Given that CD68+C1Q+ macrophages will be observed inside atretic follicles, but not in healthy growing antral follicles (Figure 1C), the follicles containing those CD68+ cells may therefore be undergoing atresia. To identify the atretic follicles containing activated immune cells, we calculated the ratio of the sum expression of *CD68*, *FCER1G, C1QA,* and *HLA-DRA* per total gene expression per cell (Figure 2H). After filtering the cells with a sum expression ratio above 0.001, we obtained seven cells belonging to two separate antral follicles, AF8.9 and AF8.10 (Figure 2H), that we labeled atretic. Note that those follicles did not contain an oocyte (Figure 2I).

### 2.2. Transcriptional Analysis of Oocytes from Human Small Antral Follicles

Next, we analyzed the molecular signature of the ten oocytes (CL3), presumably all in GV stage, as during collection no polar bodies were observed. To confirm the GV signature, we used two published single-cell transcriptomics datasets where human oocytes in GV, MI, and MII stages, after ovarian stimulation, were collected and sequenced [24,25]. In total, we retained 7 GV, 7 MI, and 24 MII oocytes for analysis after excluding 1 MII oocyte due to high percentage of mitochondrial genes (Supplementary Figure S3A). Using a list of known markers for oocytes and cumulus GCs (cGCs) [3], we observed that six oocytes (five from published datasets and one from our antral follicle AF2.4) showed high expression of cGC markers and may have been contaminated by cGCs (Supplementary Figure S3B). Those six oocytes were excluded from further analysis. 

**Figure 3:**
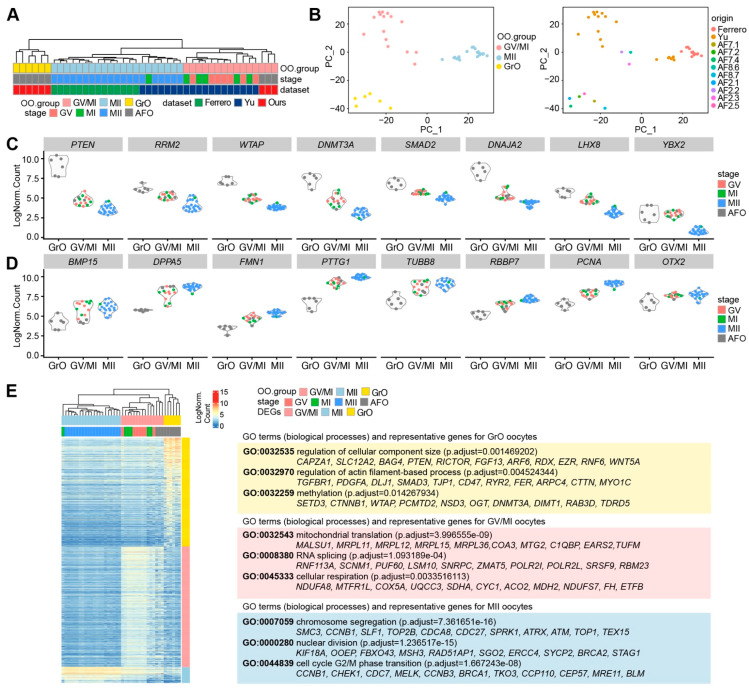
Transcriptional analysis of oocytes from antral follicles. (**A**) Hierarchical clustering of oocytes from antral follicles (AFO) and oocytes in GV, MI, and MII stage from published datasets, based on the 100 most highly variable expressed genes; (**B**) Principal Component Analysis (PCA) of AFO, GV, MI, and MII oocytes colored by oocyte groups (left panel) and oocyte origins (right panel); (**C**) Violin plots of selected genes showing decreasing expression from the GrO to the MII group; (**D**) Violin plots of selected genes showing increasing expression from the GrO to the MII group; (**E**) Heatmap showing differentially expressed genes in the OO.groups and several representative GO terms (biological processes) and associated genes (see Supplementary Table S2).

Next, we performed unsupervised hierarchical clustering of the retained oocytes, including our nine (antral follicle) oocytes (AFOs), using the 100 most highly variable expressed genes, and obtained three main branches (OO.group): one with MII oocytes that underwent ovarian stimulation (MII group); one with the GV and MI oocytes that underwent ovarian stimulation as well as three AFOs (GV/MI group); and one with six AFOs (GrO group) (Figure 3A). Similar to the hierarchical clustering, principal component analysis (PCA) separated the MII group from the rest on the first principal component (PC1); whereas the GV/MI group separated from the GrO group on the second principal component (PC2) (Figure 3B), suggesting that the GV and MI oocytes have a relatively similar molecular signature. To investigate whether the oocytes in the GrO group were of low quality, we plotted the number of total counts, number of RNA features, and percentage of mitochondrial RNA on the PCA plot (Supplementary Figure S3C), but that did not show signs of low quality.

Genes of interest that were prominently expressed in the GrO oocytes and downregulated in GV/MI and MII oocytes included *PTEN*, *RRM2*, *WTAP*, *DNMT3A*, *SMAD2*, *DNAJA2*, *LHX8,* and *YBX2* (Figure 3C). Additionally, genes of interest that gradually increased from GrO to MII oocytes, included *BMP15*, *DPPA5*, *FMN1*, *PTTG1*, *TUBB8*, *RBBP7*, *PCNA,* and *OTX2* (Figure 3D), all reported to be highly expressed in human mature oocytes [25,26,27]. This suggested that GrO oocytes may be less mature than GV oocytes.

We calculated the DEGs for each OO.group (p_val_adj < 0.01 and Log2FC > 2) and calculated the associated Gene Ontology terms (Figure 3E; Supplementary Table S2). Genes highly expressed in MII oocytes related to “chromosome segregation”, “nuclear division”, and “cell cycle G2/M phase transition”. On the other hand, part of the genes upregulated in GV/MI group were associated with “mitochondrial translation”, “RNA splicing”, and “cellular respiration”, which have been linked to oocyte maturation and competence [28,29,30]. In the GrO group, we observed terms that were related to oocyte growth and epigenetics, such as “regulation of cellular component size”, “regulation of actin filament-based process”, and “methylation” (Figure 3E).

### 2.3. Transcriptional Analysis of Granulosa Cells from Human Non-Dominant Antral Follicles

**Figure 4:**
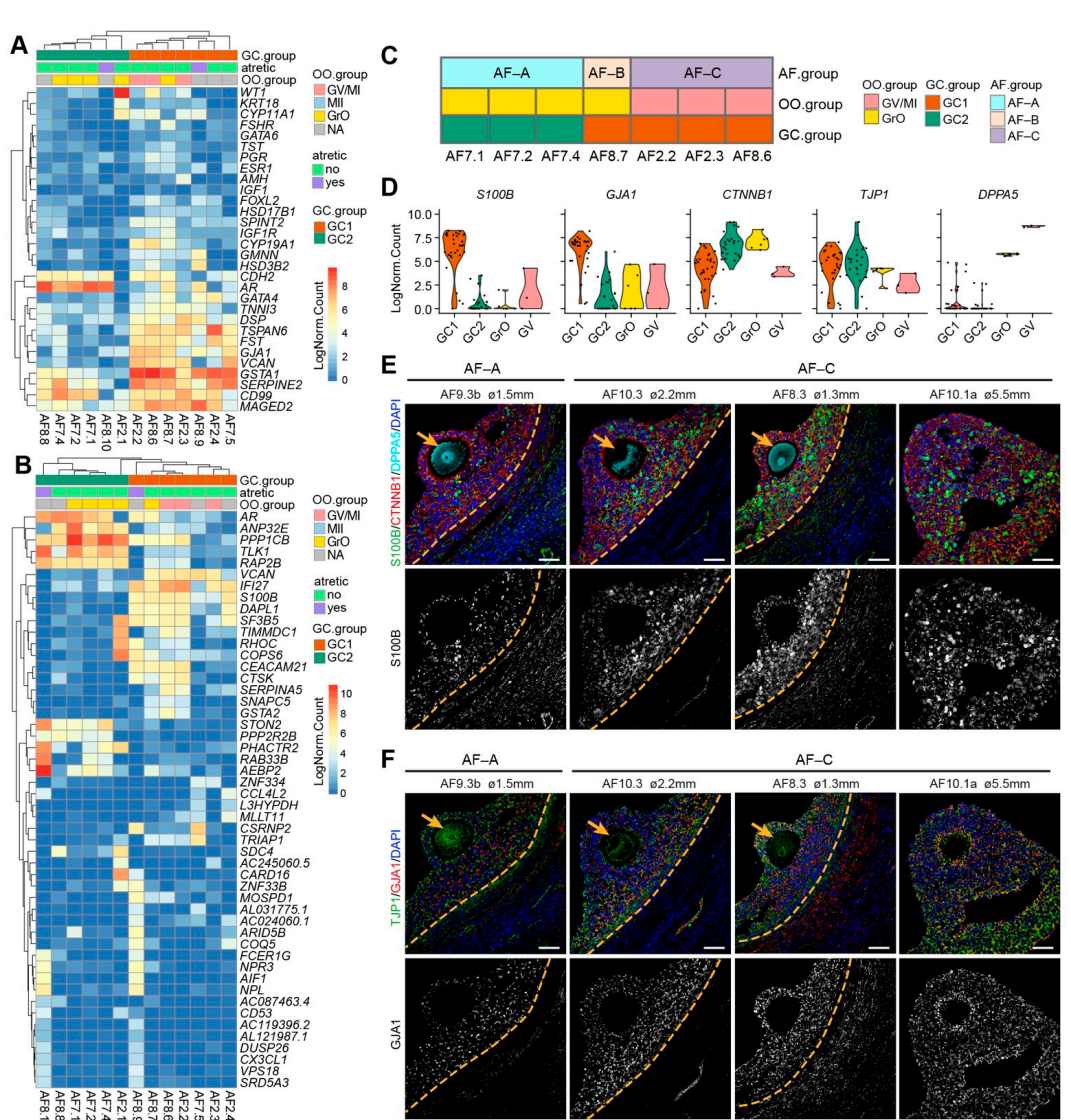
Transcriptional analysis of granulosa cells (GCs) per human small antral follicle. (**A**) Hierarchical clustering of GCs per small antral follicle based on selected GC marker genes. NA indicates follicles without oocyte or excluded oocyte; (**B**) Hierarchical clustering of GCs per small antral follicle based on the 50 most highly variable expressed genes. NA, indicates follicles without oocyte or excluded oocyte; (**C**) Summary of healthy antral follicles types identified in this study; (**D**) Violin plots showing expression of selected genes in different OO.groups and GC.groups; (**E**) Immunofluorescence for S100B, CTNNB1 and DPPA5 (top panels) and single channel for S100B (bottom panels) on small human antral follicles showing the follicle area close to the oocyte. Orange arrows indicate the oocyte. Orange dashed lines indicate the follicular basement membrane. (**F**) Immunofluorescence for TJP1 and GJA1 (top panels) and single channel for GJA1 on small human antral follicles showing the follicle area close to the oocyte. Orange arrows indicate the oocyte. Orange dashed lines indicate the follicular basement membrane. ø, follicle diameter. Scale bars are 50 μm.

The two types of oocytes present in the small antral follicles (GrO and GV, as none of the oocytes had a polar body at the time of collection), compelled us to investigate whether the matching GCs in the corresponding follicles also presented separate signatures. First, we calculated the mean gene expression of GCs (cells from CL0) per antral follicle and investigated the expression of known GC markers [3]. The mean GC expression per follicle separated the follicles in two main groups (GC1 and GC2) (Figure 4A). GCs from antral follicles with GV oocytes (AF2.2, AF2.3, AF8.6) clustered in GC1 group, which expressed higher levels of general GC markers (*VCAN*, *FST*, *GJA1*, *TNNI3, GSTA1*) and steroidogenic enzymes, such as *CYP11A1*, *CYP19A1,* and *HSD3B2* (Figure 2G and Figure 4A). The GC2 group displayed high expression of *AR* (Androgen receptor) (Figure 4A), which is upregulated in human pre-antral follicles and reaches peak in small antral follicles, being reduced in larger antral follicles [31]. We calculated the DEGs for each GC.group (p_val_adj < 0.01 and Log2FC > 2) and calculated the associated Gene Ontology terms (Supplementary Table S3).

Next, we applied unsupervised hierarchical clustering using the 50 most highly variable expressed genes, which primarily separated GC1 and GC2 (Figure 4B). Notably, GCs from the two atretic follicles (AF8.9 and AF8.10) showed high expression of genes such as *FCER1G*, *CD53*, *AIF1,* and *CX3CL1* (Figure 4B), which are associated with myeloid leukocyte activation [32,33], indicating that although the GCs from atretic follicles still express GC specific genes, they also convey signals for immune cells infiltration during atresia.

According to the signatures obtained for the oocytes and associated follicular GCs, we separated seven antral follicles into three types: type A (AF-A) contained a GrO oocyte and GC2 GCs (AF7.1, AF7.2, and AF7.4), type B (AF-B) contained a GrO oocyte and GC1 GCs (AF8.7), and type C (AF-C) contained a GV oocyte and GC1 GCs (AF2.2, AF8.6, and AF2.3) (Figure 4C). To validate the presence of the different types of follicles in the human ovarian tissue, we first identified a set of genes to distinguish GC1 from GC2 (*S100B*, *GJA1*, *CTNNB1*) and to distinguish GrO from GV (*TJP1*, *DPPA5*) (Figure 4D). From the four small antral follicles that we examined, AF9.3b showed a small number of GCs expressing high levels of S100B and lower expression of GJA1, compared with the other three follicles (AF10.3, AF8.3, AF10.1a) (Figure 4E,F). Hence, AF9.3b may represent follicle type AF-A and AF10.3, AF8.3, AF10.1a may represent follicle type AF-C.

### 2.4. Ligand–Receptor Interactions between Oocytes and GCs in Different Groups of Antral Follicles

The interaction between oocyte and GCs are decisive for follicular growth and oocyte maturation. To study the possible receptor–ligand pairs interactions between oocytes and GCs in the three types of small antral follicles, we have applied the CellphoneDB algorithm [34], filtering for *p* < 0.05 and component genes expressed in at least 30% of the cells (oocytes or GCs) per AF group (Supplementary Table S4).

**Figure 5:**
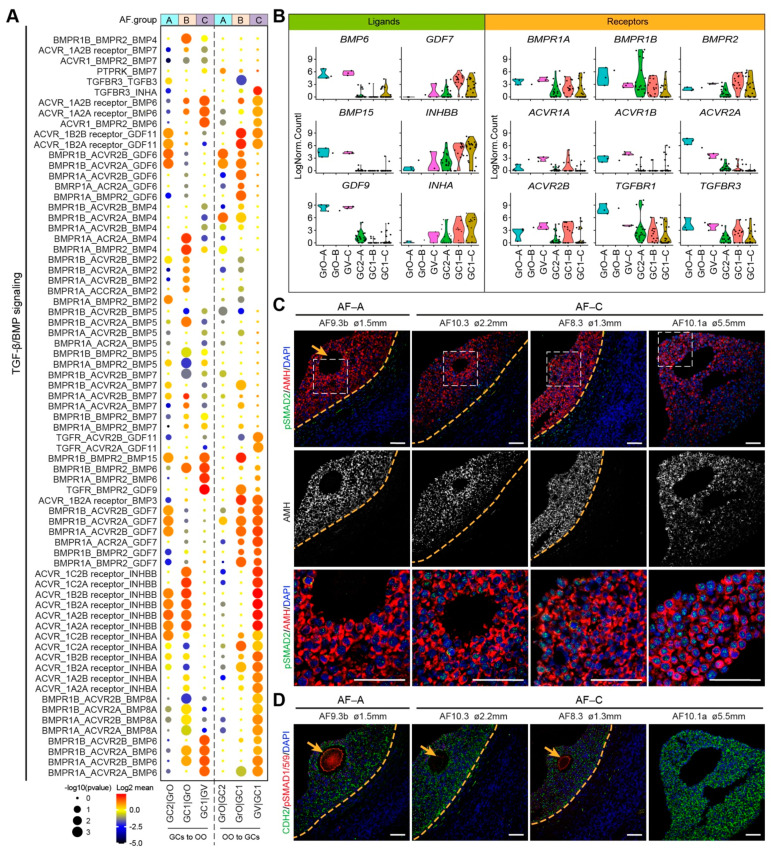
TGF-β/BMP signaling ligand–receptor interactions in different types of small antral follicles. (**A**) CellphoneDB analysis of TGF-β/BMP signaling interactions between oocytes and GCs in different types of antral follicles; (**B**) Violin plots of selected TGF-β/BMP signaling ligand and receptor genes in OO.groups and GC.groups; (**C**) Immunofluorescence for pSMAD2 and AMH (top panels) and single channel for AMH (middle panels) on small human antral follicles showing the follicle area close to the oocyte. White dashed boxes are showed in a high magnification in the bottom panel. Orange arrows indicate the oocyte. Orange dashed lines indicate the follicular basement membrane; (**D**) Immunofluorescence for pSMAD1/5/9 and CDH2 on small human antral follicles showing the follicle area close to the oocyte. Orange arrows indicate the oocyte. Orange dashed lines indicate the follicular basement membrane. ø, follicle diameter. Scale bars are 50 μm.

The transforming growth factor-β (TGF-β) superfamily have been shown to have an essential role in folliculogenesis [35,36]. We analyzed receptor–ligand pairs from the TGFβ/BMP signaling pathways in the three types of antral follicles (Figure 5A). In the AF-C (GC1 and GV oocyte), we observed high expression of BMP receptor type II (*BMPR2*) in the GCs and high expression of *BMP6*, *BMP15, GDF9* in oocytes, whereas Activin receptors were expressed in oocytes and *GDF7, INHBB,* and *INHA* in GCs (Figure 5B). In agreement, BMP15/GDF9-BMPR2 interaction has been described in the ovary, preventing GC apoptosis and promoting oocyte developmental competence [37,38]. In addition, the expression of Inhibin-A (heterodimer of INHBB and INHA) in GCs has been shown to increase in antral follicles in response to follicle stimulating hormone (FSH) [39]. Together, this suggested that AF-C follicles are more mature than AF-B or AF-A. To examine the activation of TGFβ/BMP signaling pathways in AF-A and AF-C, we performed immunostaining for phosphorylated (p)SMAD2 (Figure 5C) and pSMAD1/5/9 (Figure 5D). In agreement, we observed nuclear pSMAD2 in GCs, in particular in AF-C (GC1 GCs), suggesting an active TGFβ signaling, whereas (nuclear) pSMAD1/5/9 was not observed in GCs, but staining was detected in the oocytes.

**Figure 6:**
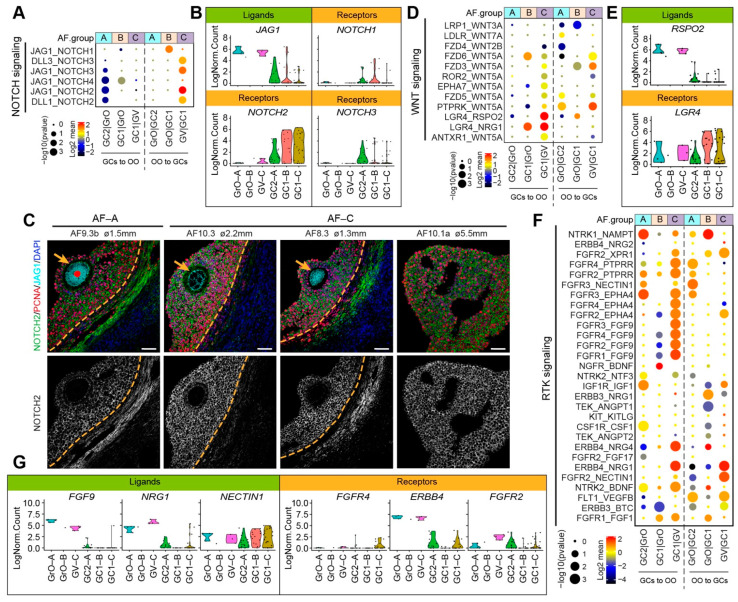
NOTCH, WNT, and RTK signaling ligand–receptor interactions in different types of small antral follicles. (**A**) CellphoneDB analysis of NOTCH signaling interactions between oocytes and GCs in different types of antral follicles; (**B**) Violin plots of selected NOTCH signaling ligand and receptor genes in OO.groups and GC.groups; (**C**) Immunofluorescence for NOTCH2, JAG1, and PCNA (top panels) and single channel for NOTCH2 (bottom panels) on small human antral follicles showing the follicle area close to the oocyte. Orange arrows indicate the oocyte. Orange dashed lines indicate the follicular basement membrane. ø, follicle diameter. Scale bars are 50 μm; (**D**) CellphoneDB analysis of WNT signaling interactions between oocytes and GCs in different types of antral follicles; (**E**) Violin plots of selected WNT signaling ligand and receptor genes in OO.groups and GC.groups; (**F**) CellphoneDB analysis of RTK signaling interactions between oocytes and GCs in different types of antral follicles; (**G**) Violin plots of selected RTK signaling ligand and receptor genes in OO.groups and GC.groups.

The NOTCH signaling pathway has also been shown to play a role in the crosstalk between oocyte and GCs during folliculogenesis [20]. In our data set, the JAG1-NOTCH2 was the most significant ligand–receptor interaction occurring in AF-C, with *JAG1* being expressed in GrO and GV oocytes, but *NOTCH1* mostly expressed in GC1 GCs (Figure 6A,B). By immunostaining, we confirmed that JAG1 was expressed in GrO and GV oocytes and that NOTCH2 was expressed in GCs, albeit less strongly in GCs from AF-A as expected (Figure 6C).

Regarding the WNT signaling pathway, we observed that interactions through *LGR4* were prominent in AF-C (Figure 6D). The R-spondin (RSPO) family have been identified as ligands of LGR4 [40], and interestingly, secretion of Rspo2 by the oocyte has been shown to activate Wnt/Ctnnb1 signaling in the GCs during early follicular growth in mice [41]. In humans, *RSPO2* was expressed in GrO and GV oocytes, but *LGR4* is predominantly expressed in GC1 GCs (Figure 6E).

Finally, we focused on the receptor tyrosine kinase (RTK) mediated signaling, which involves growth factors, such as IGFs, FGFs, NRGs, VEGFs (Figure 6F). From the significant ligand–receptor pairs, we observed interactions involving the expression of *FGF9* in oocytes and FGF receptors in GCs, particularly in AF-C (Figure 6F). Moreover, the expression of receptor–ligand pairs *NRG4/1* in oocytes and *ERBB4* in GCs as well as *NECTIN1* in GCs and *FGFR2* in oocytes were detected in AF-C follicles (Figure 6F,G). Concluding, our ligand–receptor analysis indicated that different types of small antral follicles, with different oocyte and GC signatures, also showed distinct intercellular signaling transduction activities.

## 3. Discussion

Our work is the first to investigate the transcriptional signatures of oocytes and surrounding GCs in individual human small antral follicles. We have revealed that two types of oocytes (GV and GrO) could be found in small antral follicles, but also highlighted the heterogeneity of GCs surrounding the oocyte in the cumulus area. Previous studies on the molecular characterization of GCs from small antral follicles either pooled cells of the ovary or pooled cells of the same antral follicle [9,18,20,42,43]. Here, we have analyzed both the oocyte and GCs from the same small antral follicle, by transcriptomics and immunofluorescence, and we were able to validate two different follicular signatures (AF-A and AF-C). Complementary research on follicular atresia and dominance will significantly contribute to broaden our understanding of human folliculogenesis.

It is well established that a LH peak triggers resumption of meiosis in ovulatory follicles, but that the oocytes in the (adjacent) growing follicles present in the same ovary should remain in the GV stage (or undergo atresia). However, during the procedure of ovarian tissue cryopreservation (without ovarian stimulation) oocytes in MII stage have been found during tissue preparation in both prepubertal [44] and pubertal patients [1], probably resulting from spontaneous activation during the procedure. In our study, neither MI nor MII oocytes were found during collection, as confirmed by transcriptional analysis. 

Oocytes in GV, MI, and MII stages can be distinguished by morphology (number of polar bodies), however in the data set analyzed, we and others [25] were unable to distinguish GV and MI oocytes regarding their molecular signature. This could result from the low number of oocytes analyzed but could also result from the fact the MI stage is a transitory intermediate stage between the arrested oocytes in GV and arrested oocytes in MII. Moreover, oocytes obtained from primordial to early antral follicles, all supposedly in GV stage, have been shown to be in distinct transcriptional states associated with primordial, primary, secondary, and antral follicles [20]. Our analysis identified two types of oocytes (GrO and GV), reflecting slightly different maturation states in small antral follicles.

The small data size is the main limitation of our study. Nevertheless, we provided evidence for cellular heterogeneity in the GCs located in the region of the oocyte, reflecting ongoing differentiation to cumulus and mural GCs in small antral follicles. For example, the dynamic expression pattern of pKRT and CYP19A1 in GCs was associated with cumulus/mural differentiation in the region close to the oocyte. After we pooled the GCs by follicle, we identified two groups of GC signatures in healthy small antral follicles. However, to identify the subtypes of GCs in a systematic way, more GCs from a larger number of follicles is needed.

From the antral follicles used for transcriptomics, we noticed that follicle type AF-A (GrO oocyte and GC2 GCs) only contained follicles from patient P7, which had sickle cell disease (SCD). Notably, the AF-A follicle (AF9.3b), identified by immunostaining, was collected from patient P9 that was also diagnosed with SCD. It is still unclear whether SCD results in sub-fertility; however, lower ovarian reserve and lower levels of AMH has been reported in the serum of SCD patients [45,46]. Our results suggested an impact of SCD on GC identity and oocyte-GC communication.

The bi-directional communication between oocyte and the surrounding GCs regulates both follicle growth and oocyte maturation [47]. Other than direct cell–cell communication through gap junctions, paracrine signaling also plays an important role in crosstalk between oocyte and GCs [48]. TGF-β/BMP signaling pathway is perhaps the best studied pathway during folliculogenesis [49,50]. BMP15 and GDF9 are oocyte-specific and essential for fertility [50]. The intercellular transducers of the TGF-β/BMP signaling pathway, pSMAD2/3 and pSMD1/5/8 (SMAD8 is also known as SMAD9), respectively, have been reported in GCs in mice and sheep [51,52,53]. Intriguingly, we showed presence of pSMAD1/5/9 in oocytes, which might indicate an autocrine activity of oocyte-secreted factors during follicle growth.

## 4. Materials and Methods

### 4.1. Collection of Human Ovarian Tissue

Small antral follicles used in this study were collected from 6 female patients of reproductive age (P2, P3, P7, P8, P9, P10), undergoing fertility preservation procedures. From 6 patients, 3 were oncological patients, 2 were diagnosed with sickle cell disease, and 1 with beta-thalassemia. Ovaries from patients P2, P8, and P10 were in the follicular phase, while from P3, P7, and P9 were in the luteal phase (contained a corpus luteum). Small antral follicles (*n* = 6) from P3, P8, P9, and P10 were used for immunostaining and a total of 14 antral follicles from P2, P7, and P8 were used for single-cell RNA-sequencing. For single-cell RNA-sequencing, the small antral follicles (1–8 mm) were collected in saline solution (0.9% NaCl, Fresenius Kabi, Amesfoort, Netherlands). The follicles were mechanically ruptured with tungsten needles and the region with the cumulus GCs and oocyte was trimmed and transferred into a clean dish. Individual follicular cells were manually picked up using a pulled glass-capillary under a stereo microscope (Zeiss, Oberkochen, Germany). Oocytes were denuded by pipetting up and down. None of the collected oocytes contained a polar body. Each cell was transferred into a cold PCR tube contain 1.9 μL 0.2% TritonX-100 in DEPC water and 0.1 μL Recombinant RNase inhibitor (2313A, 40U/μL, TaKaRa, Kusatsu, Japan), snap-frozen on dry ice, and stored at −80 °C.

### 4.2. Single-Cell RNA Sequencing and Data Preparation

Single-cell full-length cDNA libraries using the Smart-seq2 were prepared as previously described [54] and sequenced at the LUMC sequencing facility, using a Nextera XT DNA Library Preparation Kit (Illumina, San Diego, CA, USA) and an Illumina HiSeq 4000 and NovaSeq 6000 sequencing system (Illumina). Paired-end reads were processed using BioWDL RNA-seq pipeline [55], developed at the LUMC. In short, the pipeline first performed QC on the raw reads using cutadap (v2.10), then aligned the reads to the GRCh38 human genome using STAR (v2.7.5a). An average of 65.5% of the reads per cell were mapped to the reference genome. Next, HTSeq (v0.12.4) was used to count the reads aligning to each of the genes and produce the final cell to gene count matrix.

### 4.3. Sequencing Data Analysis

The count matrix was analyzed with a Seurat based workflow (v4.0.3) [21] using R (v4.1.0). Cells with a number of reads less than 20,000 (nCount_RNA < 20,000), a number of expressed genes less than 1000 (nFeature_RNA < 1000), and a percentage of expressed mitochondrial transcripts more than 30% were considered low quality and excluded from further analysis. The raw counts were normalized by NormalizeData function (scale.factor = 10^6^) from Seurat. Next, the top 1000 highly variable features (genes) were selected with FindVariableFeatures function. Principal Component Analysis (PCA) was performed using these top 1000 genes. The first 11 PCA dimensions were used to calculate cell clusters and project the cells on a two-dimensional plot using Uniform Manifold Approximation and Projection (UMAP) algorithm. After observing a batch effect on cell clustering and UMAP projection, the function fastMNN from batchelor package (v1.6.0), with adjusted parameters (*k* = 5) was used to correct it. New cell clustering and UMAP projection were calculated based on the batch corrected components. 

For the hierarchical clustering, the top most variable genes were selected using R function rowVars from package genefilter (v1.72.1) [56]. Differentially expressed genes (DEGs) for each cluster were calculated with function FindAllMarkers (only.pos = TRUE) from Seurat with the Wilcoxon Rank Sum test method. For GO term enrichment analysis, we first used function FindMarkers in DESeq2 to calculate DEGs between groups, followed by enrichGO function from the DOSE package (v3.14.0) [57] and the GO terms were called specifically for biological process.

### 4.4. Dataset Integration for Oocyte Transcriptional Analysis

The raw data (sequencing reads) of 7 GV, 7 MI, and 7 MII oocytes from Yu and colleagues [25] were downloaded from Gene Expression Omnibus (GEO) database with accession number PRJNA508772. The raw data of 18 MII oocytes (collected from healthy donors) from Ferrero and colleagues [24] were downloaded with accession number PRJNA514416. The BioWDL pipeline was applied to get the count matrix as described above for the in-house dataset. Later, Seurat objects were generated based on count matrixes of the two published datasets and then merged with count matrix of oocytes from in-house dataset for further analysis.

### 4.5. Receptor–Ligand Interaction Analysis

The CellphoneDB algorithm (v2.1.2) [34] was used to obtain receptor–ligand pairs for analyzing interactions between oocyte and GCs. Normalized count data was extracted from Seurat object and used as input. Receptor–ligand pairs were called independently for oocytes and GCs from the 3 types of follicles. The receptor–ligand pairs involved in the specific signaling pathways were selected as previously described [58]. After filtering for *p* < 0.05, the retained receptor–ligand pairs with component genes expressed in more than 30% oocytes or GCs per antral follicle group. In brief, the receptor–ligand pairs selected for TGFβ/BMP signaling were filtered by key (partial) gene symbols including *ACVR, BMP, INHBA, INHBB, AMH, MIS, TGF,* and *GDF*; *NOTCH* was used for NOTCH signaling; *WNT, FZD, RSPO, LGR, LRP5,* and *LRP6* were used for WNT signaling; *CSF1R, EGFR, EPHA2, ERBB2, ERBB3, ERBB4, FGFR, FGFR2, FGFR3, FGFR4, FLT1, FLT3, FLT4, IGF1R, INSR, KDR, KIT, MET, NGFR, NTRK1, NTRK2, PDGFRA, PDGFRB,* and *TEK* were used for RTK signaling. The dot_plot function from CellphoneDB was applied to visualize the results.

### 4.6. Immunofluorescence and Imaging

Human ovarian tissue was fixed overnight in 4% paraformaldehyde (PFA) at 4 °C and then embedded in paraffin on a Shandon Excelsior tissue processor (Thermo Fisher Scientific, Waltham, MA, USA). Tissue sections (5 μm thickness) were deparaffinized in xylene and rehydrated in ethanol with a sequential dilution and distilled water, the sections were then transferred into 0.01 M sodium citrate buffer (pH 6.0) and heated for 12 min at 98 °C in a TissueWave 2 Microwave (Thermo Fisher Scientific, Waltham, MA, USA). To prevent non-specific antibody binding, tissue sections were treated with 1% BSA-0.05% Tween 20-PBS buffer for 1 h at room temperature (RT), followed by overnight incubation with primary antibodies at 4 °C. The primary antibodies used were mouse anti-PCNA (1:100, SC-56, Santa Cruz, Santa Cruz, CA, USA), mouse anti-CD68 (1:50, M087629-2, DAKO, Glostrup, Denmark), rabbit anti-C1Q (1:200, A0136, DAKO), goat anti-FOXL2 (1:250, ab5096, Abcam, Cambridge, United Kingdom), mouse anti-StAR (1:100, sc166821, Santa Cruz), rabbit anti-Collagen Type IV (1:50, AB748, Merck, Kenilworth, NJ, USA), mouse anti-CYP19A1 (1:50, sc-374176, Santa Cruz), mouse anti-Cytokeratin (1:100, M351501, DAKO), mouse anti-Connexin-43/GJA1 (1:50, 13–8300, Zymed, San Francisco, CA, USA), rabbit anti-S100B (1:100, AB52642, Abcam), mouse anti-β-Catenin/CTNNB1 (1:200, 610154, BD Biosciences, Franklin Lakes, NJ, USA), goat anti-DPPA5 (1/100, AF3125, R&D Systems, Minneapolis, MN, USA), rabbit anti-ZO-1/TJP1 (1:100, 61–7300, eBioscience, Hatfield, UK), mouse anti-AMH (1:30, MCA2246T, BioRad, Hercules, CA, USA), mouse anti-N-Cadherin/CDH2 (GC-4) (1:100, C3865, Sigma-Aldrich, St. Louis, MO, USA), rabbit anti-phospho-SMAD2 (Ser465/467) (1:200, 3108, Cell Signaling, Danvers, MA, USA), rabbit anti-phospho-Smad1 (Ser463/465)/Smad5 (Ser463/465)/Smad9(Ser465/467) (1:200, 13820, Cell Signaling), rabbit anti-NOTCH2 (1:200, 5732, Cell Signaling), goat anti-JAG1 (1:50, sc-6011, Santa Cruz). Tissue sections were then washed 3 times (2 times with PBS and 1 time with 0.05% Tween 20-PBS) and incubated with secondary antibodies and DAPI (Life Technologies, Carlsbad, CA, USA) for 1 h at RT. The secondary antibodies used were Alexa Fluor 488 donkey anti-rabbit IgG (1:500, A-21206, Life Technologies), Alexa Fluor 594 donkey anti-mouse IgG (1:500, A-21203, Life Technologies), and Alexa Fluor 647 donkey anti-goat IgG (1:500, A-21447, Life Technologies). The In Situ Cell Death Detection Kit (FITC) (11684817910, Sigma-Aldrich) was used to stain for TUNEL according to the manufacturer’s instruction. ProLong Gold (Life Technologies) was used to mount the sections. Immunofluorescence was imaged on an inverted confocal microscope (SP5 CLSM, Leica, Wetzlar, Germany) with LAS software (Leica, Wetzlar, Germany). ImageJ software [59] was used for image analysis. 

### 4.7. Statistics

To calculate the DEGs for each cluster, we used the Wilcoxon rank sum test and p-value was adjusted with a Bonferroni correction (default setting from Seurat workflow). Statistics associated with the GO enrichment (using DESeq2) and the CellphoneDB analysis were integrated in the respective algorithms [34,60].

## 5. Conclusions

In conclusion, our transcriptional analysis on human small antral follicles highlighted the heterogeneity of GCs proximal to oocyte, the diversity of molecular signatures in different follicles, and the importance to study oocyte-granulosa crosstalk based on individual follicles. 

## Data Availability

RNA-sequencing data is in will be deposited in Gene Expression Omnibus (GEO) with accession number GSE186504. https://www.ncbi.nlm.nih.gov/geo/query/acc.cgi?acc=GSE186504.

## Supplementary Materials

The following are available online at https://www.mdpi.com/article/10.3390/ijms222111955/s1.
