# Supplementary material for: Single-Cell Transcriptomics Analysis of Human Small Antral Follicles
Source: Int J Mol Sci. 2021 Nov 4;22(21):11955. doi: 10.3390/ijms222111955 (PMC8584910; doi:10.3390/ijms222111955)
Supplement: Supplementary file 1 [file ijms-22-11955-s001.zip › fig suppl-R2.pdf]

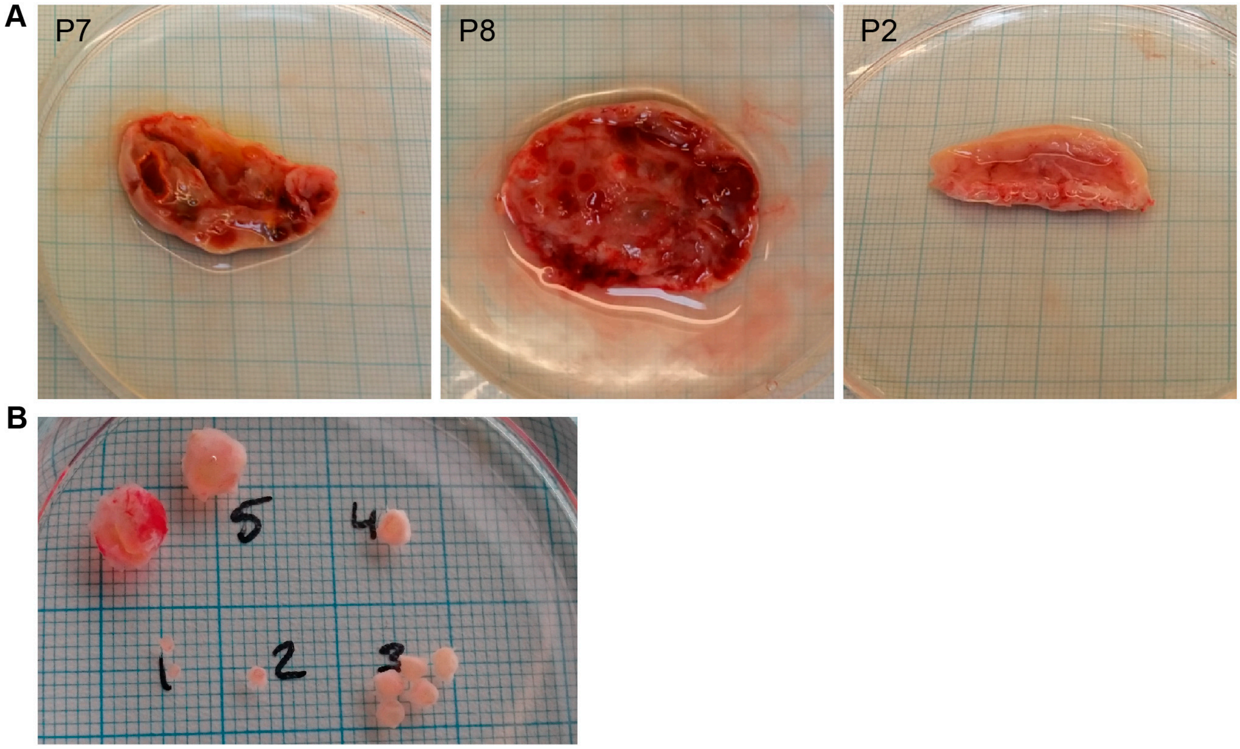

Figure S1. Human ovaries and small antral follicles used in this study. (**A**) Pictures of ovarian tissue from the 3 donors (P7, P8, P2), from which we collected cells for single-cell RNA sequencing; (**B**) Examples of fresh small antral follicles collected from donor P3.

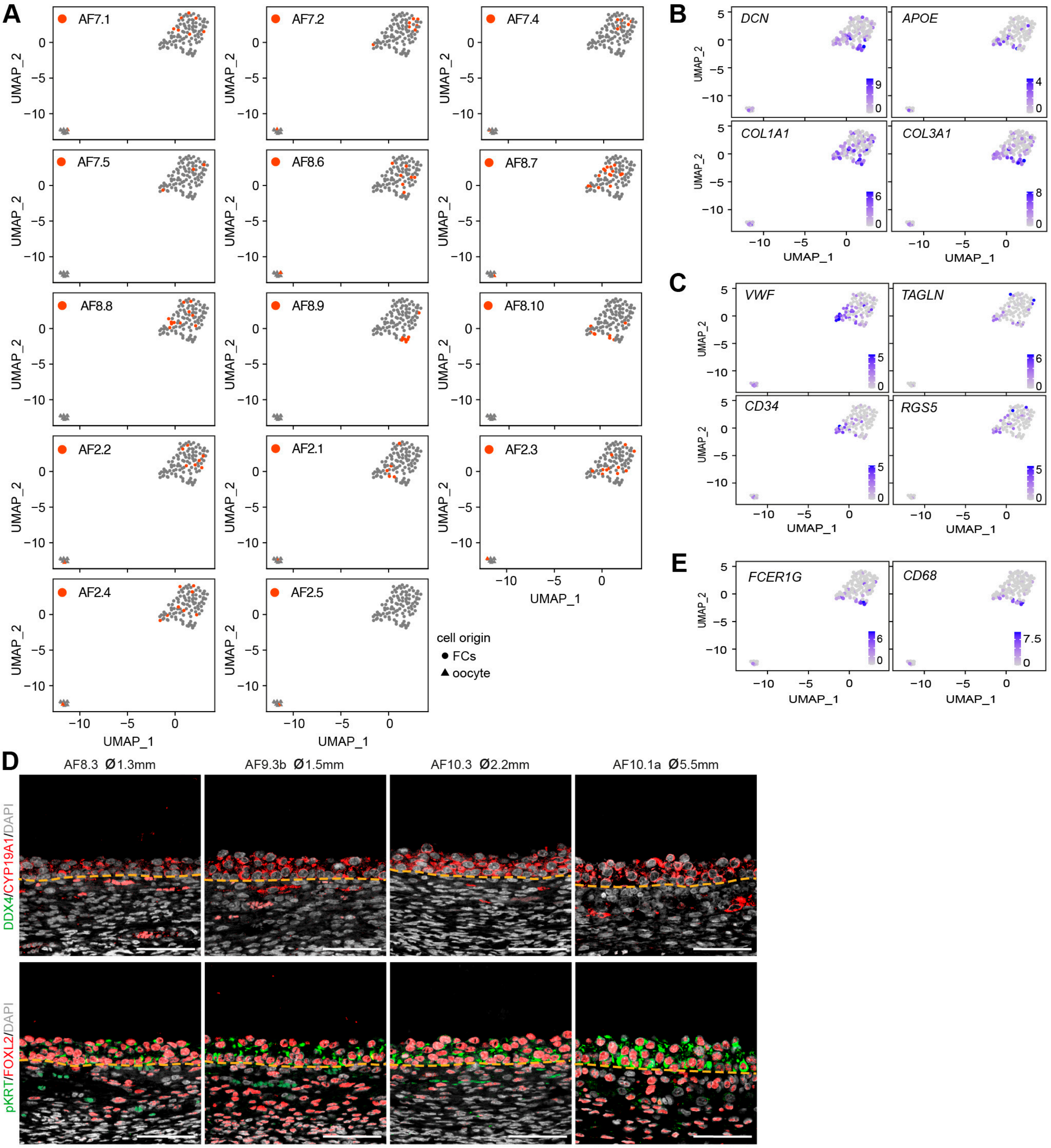

Figure S2. Identification of cells from different human antral follicles. (**A**) UMAP plots showing distribution of cells from each antral follicle; (**B**) Expression of selected ovarian stromal marker on the UMAP plot; (**C**) Expression of selected endothelial and smooth muscle markers on the UMAP plot; (**D**) **(G)** Immunofluorescence for DDX4 and CYP19A1 (top panels); and pan-cytokeratin (pKRT) and FOXL2 (bottom panels) on small human antral follicles showing the follicle area with mural granulosa cells. Orange dashed lines indicate the follicular basement membrane. ø, follicle diameter. Scale bars are 50 µm; (**E**) Expression of selected immune markers on the UMAP plot.

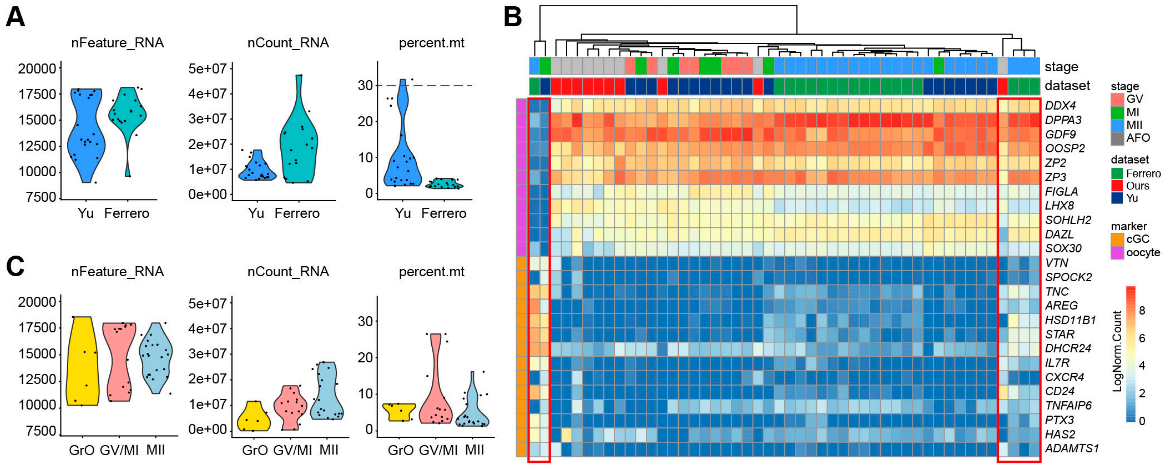

Figure S3. Quality control of oocytes used in this study. (**A**) Violin plots showing the number of features, number of total transcripts and percentage of expressed mitochondrial transcripts in the oocytes from 2 published datasets. 1 oocyte above the red dashed line was excluded from further analysis; (**B**) Heatmap showing expression of known markers for oocyte and cumulus GC in all oocytes; (**C**) Violin plots showing the number of features, number of total transcripts and percentage of expressed mitochondrial transcripts for each OO.group.
